# Methicillin-Sensitive *Staphylococcus aureus* CC398 in Intensive Care Unit, France

**DOI:** 10.3201/eid2009.130225

**Published:** 2014-09

**Authors:** Anne-Sophie Brunel, Anne-Laure Bañuls, Hélène Marchandin, Nicolas Bouzinbi, David Morquin, Estelle Jumas-Bilak, Philippe Corne

**Affiliations:** Institut de Recherche pour le Développement, Montpellier, France (A.-S. Brunel, A.-L. Bañuls, N. Bouzinbi, D. Morquin, P. Corne);; Centre Hospitalier Universitaire de Montpellier, Montpellier (A.-S. Brunel, H. Marchandin, E. Jumas-Bilak, D. Morquin, P. Corne);; Université Montpellier 1, Montpellier (H. Marchandin, E. Jumas-Bilak)

**Keywords:** methicillin-sensitive Staphylococcus aureus, MSSA, bacteria, clonal complex 398, intensive care unit, multilocus sequence typing, double-locus sequence typing, agr typing, spa typing, molecular epidemiology, France

## Abstract

During testing for *Staphylococcus aureus* in an intensive care unit in France in 2011, we found that methicillin-sensitive *S. aureus* clonal complex 398 was the most frequent clone (29/125, 23.2%). It was isolated from patients (5/89, 5.6%), health care workers (2/63, 3.2%), and environmental sites (15/864,1.7%). Results indicate emergence of this clone in a hospital setting.

Livestock-associated methicillin-resistant *Staphylococcus aureus* (MRSA) sequence type (ST) 398, which belongs to clonal complex (CC) 398, is an emergent zoonotic agent responsible for massive colonization of livestock and food products and infections in humans worldwide (*1*). Recently, emergence of animal-independent methicillin-sensitive *S. aureus* (MSSA) ST398 has been reported in China (*2*), France (*3**,**4*), the Netherlands (*5**,**6*), Spain (*7*), and North America (*8**–**11*). MSSA ST398 has been reported in colonized (*5**,**7**,**8**,**10**,**11*) and infected (*2**,**5**,**6**,**8**,**10**,**11*) patients. These isolates have been characterized as having staphylococcal protein A (spa) type t571, being sensitive to all antimicrobial drugs except macrolides, and having variable presence of Panton-Valentine leukocidin (*2**,**3**,**8*). In France, an increasing incidence of MSSA ST398 bacteremia has been observed since 2007 (*3**,**4*).

During a systematic, molecular, epidemiologic survey of *S. aureus* in an intensive care unit (ICU) in France, *S. aureus* CC398 was isolated from patients, health care workers (HCWs), and environmental sites. We conducted a study to describe the spread and characteristics of *S. aureus* CC398 in this setting.

## The Study

A prospective molecular epidemiologic study of *S. aureus* was performed in a 12-bed ICU at the University Hospital in Montpellier, France, during 5 months in 2011. *S. aureus* nasal carriage was investigated at admission and weekly in 89 patients and monthly in 63 volunteer health care workers (HCWs). Simultaneously, all *S. aureus* isolates from clinical samples were obtained from the hospital laboratory of bacteriology and clinical data were recorded.

Pneumonia was diagnosed on the basis of clinical, biologic, and radiologic criteria, and a colony count ≥10^4^ CFU/mL in bronchoalveolar fluid culture or ≥10^7^ CFU/mL in sputum cultures. Bronchial colonization was defined as a colony count <10^7^ CFU/mL in sputum cultures in asymptomatic patients.

Random sampling of surfaces was performed monthly in all rooms of the ICU (864 environmental sites). Isolates were characterized by using multilocus sequence typing, double-locus sequence typing (DLST), and accessory gene regulation (agr) typing. Resistance to antimicrobial drugs was detected by using the disk-diffusion method. Virulence genes and *ermA*, *ermC*, *ermT*, and *msrA* genes were screened for by using PCRs.

**Figure 1:**
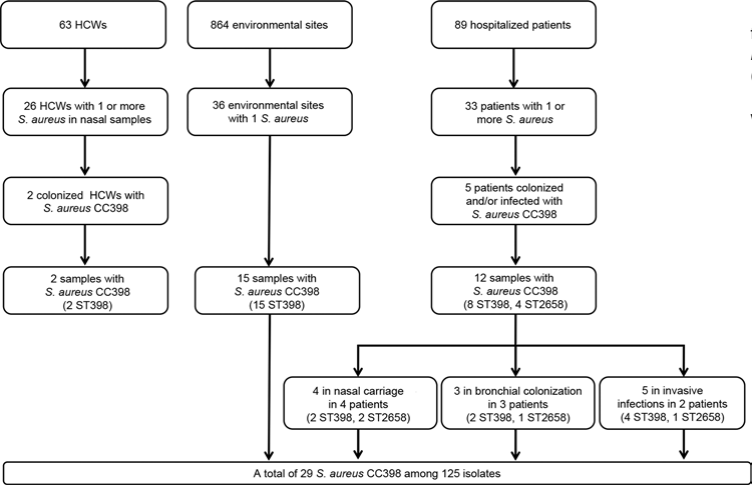
Flowchart of selection for methicillin-sensitive *Staphylococcus aureus* clonal complex (CC) 398 from intensive care unit, France, 2011. HCWs, health care workers; ST, sequence type.

**Figure 2:**
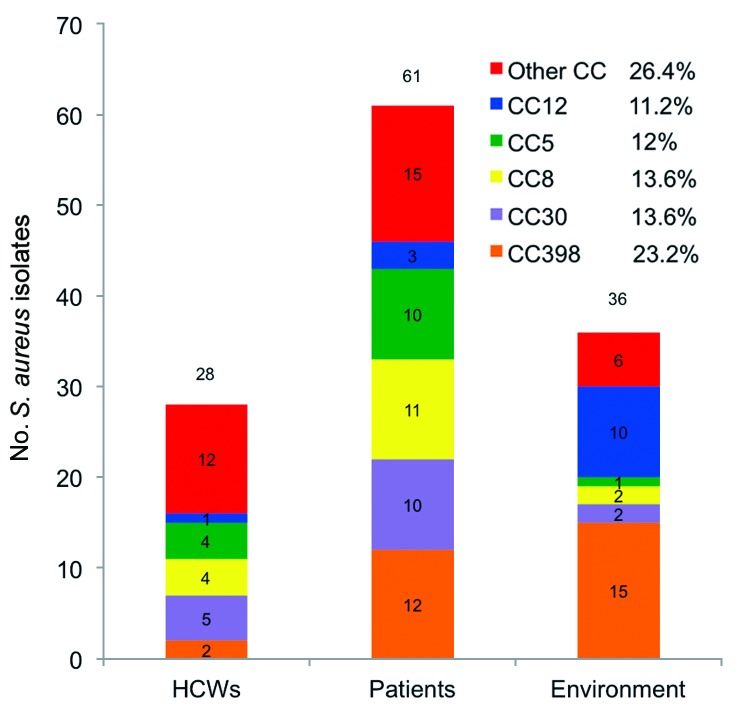
Principal clonal complexes (CCs) among 125 isolates of *Staphylococcus aureus* from intensive care unit, France, 2011. HCWs, health care workers.

During the survey period, the number of samples obtained ranged from 1 to 32 per patient and from 1 to 3 per HCW. Of these samples, 125 *S. aureus* isolates (110 MSSA and 15 MRSA) were obtained from 33 patients, 26 HCWs, and 36 environmental sites; these isolates belonged to 28 STs and 12 CCs. Among these 125 isolates, 12 isolates from 5 patients, 2 isolates from 2 HCWs, and 15 isolates from 15 environmental sites belonged to CC398 (Figure 1; Technical Appendix). The 29 strains were MSSA and belonged to ST398 (n = 25) or to a new ST submitted to the MLST Database (http://www.mlst.net/) as ST2658 (n = 4). ST398 and CC398 were the most prevalent genotype and clonal complex identified: 25/125 (20%) and 29/125 (23.2%) isolates, respectively (Figure 2).

The prevalence of MSSA CC398 carriage was 3.2% (2/63) in HCWs and 5.6% (5/89) in patients. The prevalence of MSSA CC398 infection was 2.25% (2/89 patients) (Figure 1). These patients were hospitalized during the same period; nosocomial pneumonia developed after nasal colonization, and was associated with bacteremia in 1 case. Demographic and clinical characteristics were similar in patients colonized or infected with MSSA CC398 or with other genotypes (Table 1). No history of contact with livestock was found for patients and HCWs. The prevalence of MSSA CC398 environmental contamination was 1.7% (15/864 samples). Genotype CC398 was found more frequently in the ICU environment (15/36, 41.7%) than in patients (5/33, 15.2%; χ^2^ 4.7, p = 0.03) and HCWs (2/26, 7.7%; χ^2^ 7.1, p = 0.007) (Technical Appendix).

**Table 1:** Demographic and clinical characteristics of 33 patients colonized or infected with *Staphylococcus aureus*, intensive care unit, France, 2011*

Characteristics	*S. aureus* CC398 (n = 5 patients)	Another genotype of *S. aureus* (n = 28 patients)	p value
Demographic data			
M:F ratio	5	1.5	
No. (%) men	5 (100)	17 (60.7)	0.14
Mean age, y	53.4	53.9	0.96
Concurrent conditions, no. (%)			
Diabetes	0	6 (21.4)	0.55
COPD/CRF	1 (20)	5 (17.8)	1
Cancer/hematologic disease	1 (20)	1 (3.6)	0.28
Chemotherapy/IS	0	3 (10.7)	1
HIV infection	0	0	1
Risk factors for *S. aureus* colonization, no. (%)			
Hospitalization >48 h to <1 y	2 (40)	15 (53.6)	0.66
History of *S. aureus* carriage or infection	0	7 (25)	0.56
Antimicrobial drug therapy for <3 mo	2 (40)	13 (46.4)	1
Residence in long-term care facility	0	0	1
Surgery/invasive procedure within 1 y	2 (40)	7 (25)	0.61
Chronic skin wounds	0	2 (7.1)	1
Colostomy	0	0	1
Indwelling urinary catheter	0	1 (3.6)	1
Tracheotomy	0	1 (3.6)	1
Medical data related to ICU, no. (%)			
Median length of ICU stay, d	38.4	13	0.09
Mechanical ventilation	5 (100)	16 (57)	0.13
Median length of invasive ventilation , d	33.8	18.3	0.14
Severity score on admission (SAPS II)	41.6	37.6	0.64
Use of vasoactive drugs	3 (60)	13 (46.4)	0.66
Extrarenal replacement	0	2 (7.1)	1
Deaths in ICU	2 (40)	5 (17.8)	0.28

*COPD, chronic obstructive pulmonary disease; CRF, chronic respiratory failure; IS, immunosuppressive therapy; ICU, intensive care unit; SAPS II, simplified acute physiology score II.

Molecular typing of CC398 strains and microbiologic results are shown in Table 2. Four strains belonged to the new ST2658, which differed from ST398 by a synonymous mutation (A→G) at position 198 of the *pta* gene. These 4 strains were isolated from nasal carriage samples (n = 2), bronchial colonization samples (n = 1), and pneumonia testing samples (n = 1) from 2 patients hospitalized at the same time. All MSSA CC398 strains were agr type 1, spa type t571 (determined by using DNAGear software; http://w3.ualg.pt/hshah/DNAGear/), and DLST type 144–186 (DLST spa 186 corresponding to spa type t571). Genes encoding Panton-Valentine leukocidin, toxic shock syndrome toxin 1, and staphylococcal enterotoxin A were not detected. Sensitivity testing of MSSA CC398 isolates showed that all isolates were resistant to erythromycin and had an inducible macrolide–lincosamide–streptogramin B phenotype. In addition, resistance to penicillin and amoxicillin caused by β-lactamase production was observed in 41.4% (12/29) of the strains. Resistance to kanamycin, tobramycin, and gentamicin was observed in 24.1% (7/29) of the strains; all 7 strains were isolated from environmental samples. Analysis of genes encoding antimicrobial drug resistance identified the *erm*T gene in all the CC398 strains and a variable distribution of *erm*A and *erm*C genes.

**Table 2:** Microbiological characteristics of 29 *Staphylococcus aureus* clonal complex 398 isolates from intensive care unit, France, 2011

Source*	Place of isolation†	Date of isolation	MLST‡	Resistance phenotype§	MLS resistance genes¶			
					*erm*A	*erm*C	*msr*A	*erm*T
P1	S	Jan 31	ST2658	BL, iMLS	–	–	–	+
P1	N	Jan 31	ST2658	BL, iMLS	–	–	–	+
P2	N	Feb 14	ST2658	BL, iMLS	–	–	–	+
P2	S#	Feb 21	ST2658	BL, iMLS	–	–	–	+
P3	N	Feb 28	ST398	BL, iMLS	–	–	–	+
P4	BAL#	Apr 6	ST398	iMLS	–	+	–	+
P4	B#	Apr 8	ST398	iMLS	–	–	–	+
P4	BAL#	Apr 8	ST398	iMLS	–	–	–	+
P4	N	Apr 11	ST398	iMLS	–	–	–	+
P4	S#	Apr 11	ST398	iMLS	–	–	–	+
P4	S	May 9	ST398	iMLS	–	–	–	+
P5	S	June 14	ST398	BL, iMLS	–	–	–	+
HCW1	N	Feb 13	ST398	BL, iMLS	–	–	–	+
HCW2	N	Feb 14	ST398	iMLS	–	–	–	+
E	HCW kitchen, microwave	Mar 2	ST398	BL, iMLS	–	+	–	+
E	Care room no. 1, telephone	Mar 2	ST398	iMLS	–	+	–	+
E	Doctor’s telephone	Apr 5	ST398	iMLS	–	+	–	+
E	HCW kitchen, lunch table	Apr 5	ST398	BL, iMLS	–	+	–	+
E	Staff room, notebook	Apr 5	ST398	BL, iMLS	–	–	–	+
E	Medical room, telephone	Apr 5	ST398	iMLSB, KTG	–	+	–	+
E	Material room, telephone	Apr 5	ST398	iMLSB, KTG	–	+	–	+
E	Refrigerator in office	Apr 6	ST398	iMLSB, KTG	–	+	–	+
E	Bedroom no. 7, table	Apr 6	ST398	iMLSB, KTG	–	+	–	+
E	Bedroom no. 8, care card	Apr 6	ST398	iMLSB, KTG	–	+	–	+
E	Bedroom no. 10, infusion manifold	Apr 6	ST398	iMLSB, KTG	–	–	–	+
E	Care room no. 3, telephone	Apr 6	ST398	iMLSB, KTG	–	–	–	+
E	Care room no. 2, furniture	Apr 6	ST398	BL, iMLS	+	–	–	+
E	Bedroom no. 1, infusion manifold	May 9	ST398	iMLS	+	–	–	+
E	Bedroom no. 12, negatoscope	May 9	ST398	BL, iMLS	–	–	–	+

*P, patient; HCW, health care worker, E, environment.  †S, sputum; N, nose; BAL, bronchoalveolar lavage fluid; B, bloodstream. ‡MLST, multilocus sequence type; ST, sequence type. §BL, β-lactamase (resistance to penicillin and amoxicillin); iMLS, inducible macrolide–lincosamide–streptomycin B; KTG, kanamycin, tobramycin, gentamicin. ¶–, negative; +, positive.  #Strains isolated from infected patients.

## Conclusions

Identification of MSSA CC398 in HCWs, patients without exposure to livestock, and the environment in an ICU indicates emergence of this clone in a hospital in France. The prevalence of nasal carriage in HCWs and patients was high (≤5.6%) in the context of the ICU, where these persons have frequent contact with each other. The small number of patients colonized or infected with *S. aureus* CC398 limits statistical comparison of the 2 groups and identification of risk factors for infection.

Despite the monocentric nature and the short period of the study, which limit extrapolation of our results to other settings, our study underlines the capacity of MSSA CC398 to circulate among and between patients, HCWs, and the ICU environment. Slingerland et al. reported prolonged survival of bovine MSSA ST398 strain in the human nose after artificial inoculation, which suggested that competition with human strains might facilitate its spread (*12*). Identification of ST2658 in 2 patients hospitalized at the same time reinforces the hypothesis of an increased capacity of transmission of this clonal complex between patients.

Person-to-person spread of MSSA ST398 has been reported within community households (*8**,**10**)* and more recently in a hospital (*11*) and an urban jail (*9*), in which a high proportion of detainees sharing a holding tank were colonized with MSSA ST398 (*9*). These findings contrast with limited transmissibility of livestock-associated MRSA ST398, which is partially explained by molecular signatures of bacterial host adaptation identified only in the MSSA ST398 genome, such as different composition of adhesion genes that result in enhanced adhesion to human skin (*10*).

All strains were spa type t571, which is the major spa type associated with MSSA ST398 (*2**,**3**,**5**–**7*). There are other similarities between our strains and strains from China, Spain, Belgium, and the United States. (*2**,**6**,**7**,**11*), such as agr type 1, the presence of the *erm*T gene, tetracycline susceptibility, and macrolide–lincosamide–streptogramin B phenotype.

In ICUs, colonized or infected patients constitute the main reservoir of *S. aureus* (*13*). The association of MSSA CC398 with the ICU environment suggests that this environment could play a role as a bacterial reservoir as described (*14*). One hypothesis for such an association is the capacity to form a biofilm, which could be correlated with the *S. aureus* genetic background (*15*). Our findings emphasize potential hospital-adapted characteristics of *S. aureus* CC398, which is supported by others studies (*6*,*11*), and indicate that surveillance programs are needed to determine the role of this clonal complex, particularly in the hospital setting.

Technical Appendix Distribution of *Staphylococcus aureus* CC398 isolates from patients in intensive care unit, health care workers, and environmental sites, France, 2011.
