# Supplementary material for: Methicillin-Sensitive Staphylococcus aureus CC398 in Intensive Care Unit, France
Source: Emerg Infect Dis. 2014 Sep;20(9):1511–5. doi: 10.3201/eid2009.130225 (PMC4178408; doi:10.3201/eid2009.130225)
Supplement: Technical Appendix — Distribution of Staphylococcus aureus CC398 isolates from patients in intensive care unit, health care workers, and environmental sites, France, 2011. [file 13-0225-Techapp-s1.pdf]

Article DOI: http://dx.doi.org/10.3201/eid2009.130225

Methicillin-Sensitive *Staphylococcus aureus* CC398 in Intensive Care Unit

Technical Appendix

Technical Appendix Table. Distribution of *Staphylococcus aureus* CC398 isolates from patients in intensive care unit, health care workers, and environmental sites, France*

Genotype	Patients (n = 33)	HCWs (n = 26)	Environmental sites (n = 36)
CC398	5	2	15†
Other	28	24	21

*CC, clonal complex; HCWs, health care workers. Patients had ≥1 sample positive for *S. aureus* CC398.
†$p < 0.05$ by χ^2 test.
